# Effectiveness and cost-effectiveness of 'BeweegKuur', a combined lifestyle intervention in the Netherlands: Rationale, design and methods of a randomized controlled trial

**DOI:** 10.1186/1471-2458-11-815

**Published:** 2011-10-19

**Authors:** Brenda AJ Berendsen, Marike RC Hendriks, Evert ALM Verhagen, Nicolaas C Schaper, Stef PJ Kremers, Hans HCM Savelberg

**Affiliations:** 1 Human Movement Science, NUTRIM, School for Nutrition, Toxicology and Metabolism, Maastricht University Medical Centre, PO Box 616, 6200 MD Maastricht, the Netherlands; 2 Department of Public and Occupational Health, EMGO+ Institute for Health and Care Research, VU University Medical Centre, Amsterdam, the Netherlands; 3 Internal Medicine, CAPHRI, School for Public Health and Primary Care, Maastricht University Medical Centre, Maastricht, the Netherlands; 4 Department of Health Promotion, NUTRIM, School for Nutrition, Toxicology and Metabolism, Maastricht University Medical Centre, Maastricht, the Netherlands

## Abstract

**Background:**

Improving the lifestyle of overweight and obese adults is of increasing interest in view of its role in several chronic diseases. Interventions aiming at overweight or weight-related chronic diseases suffer from high drop-out rates. It has been suggested that Motivational Interviewing and more frequent and more patient-specific coaching could decrease the drop-out rate. 'BeweegKuur' is a multidisciplinary lifestyle intervention which offers three programmes for overweight persons. The effectiveness and the cost-effectiveness of intensively guided programmes, such as the 'supervised exercise programme' of 'BeweegKuur', for patients with high weight-related health risk, remain to be assessed. Our randomized controlled trial compares the expenses and effects of the 'supervised exercise programme' with those of the less intensively supervised 'start-up exercise programme'.

**Methods/Design:**

The one-year intervention period involves coaching by a lifestyle advisor, a physiotherapist and a dietician, coordinated by general practitioners (GPs). The participating GP practices have been allocated to the interventions, which differ only in terms of the amount of coaching offered by the physiotherapist. Whereas the 'start-up exercise programme' includes several consultations with physiotherapists to identify barriers hampering independent exercising, the 'supervised exercise programme' includes more sessions with a physiotherapist, involving exercise under supervision. The main goal is transfer to local exercise facilities. The main outcome of the study will be the participants' physical activity at the end of the one-year intervention period and after one year of follow-up. Secondary outcomes are dietary habits, health risk, physical fitness and functional capacity. The economic evaluation will consist of a cost-effectiveness analysis and a cost-utility analysis. The primary outcome measures for the economic evaluation will be the physical activity and the number of quality-adjusted life years. Costs will be assessed from a societal perspective with a time horizon of two years. Additionally, a process evaluation will be used to evaluate the performance of the intervention and the participants' evaluation of the intervention.

**Discussion:**

This study is expected to provide information regarding the additional costs and effects of the 'supervised exercise programme' in adults with very high weight-related health risk.

**Trial registration number:**

ISRCTN: ISRCTN46574304

## Background

The increasing prevalence of overweight and obesity is a major problem in Western countries. People who are overweight are at higher risk of developing type 2 diabetes mellitus, cardiovascular disease and certain types of cancer [1]. In addition, their health-related quality of life decreases due to the overweight as such as well as to related comorbidities [2]. In the Netherlands, 42% of women and 53% of men are overweight (BMI > 25 kg/m^2^), of which 12% and 11% respectively are obese (BMI > 30 kg/m^2^) [3]. Health care expenses caused by overweight in the Netherlands amounted to half a billion Euros in 1999 [4].

Not only overweight but also physical inactivity have been associated with chronic diseases like type 2 diabetes and cardiovascular disease [5-7]. Intervening in people's lifestyles could help decrease the severity of chronic diseases and the risk of developing them. Combined lifestyle interventions aimed at increasing physical activity and improving dietary behaviour have been shown to have positive effects on metabolic and cardiovascular risk factors (e.g. weight, waist circumference, fat mass, HDL-cholesterol and triglyceride values and blood pressure) in persons at risk for developing chronic diseases [8-13], as well as in patients who have already developed type 2 diabetes [14-17]. Beneficial effects are still evident after a follow-up period of several years [7,8].

Recently, a multidisciplinary combined lifestyle intervention for type 2 diabetes patients, called 'BeweegKuur', has been developed by the Netherlands Institute for Sport and Physical Activity (NISB) [18,19]. Its target population currently also comprises overweight and obese patients. The primary goal of the 'BeweegKuur' interventions is to improve physical activity and dietary behaviour and thereby decrease health risks. A recent study reported, however, that the adherence to exercise intervention programmes varies widely, from 10% to 80% [20]. The main causes of drop-out are exercise-related injuries and motivational factors [14]. It seems likely, therefore, that the use of Motivational Interviewing [20,21] and the individualization of the 'BeweegKuur' programmes would result in lower drop-out rates. In addition, it has been proposed to have practice nurses play a key role in the adoption of long-term behavioural change by providing this individualized guidance in the primary health care setting [9,22]. In the 'BeweegKuur' programmes, the participant's behavioural change is supported by a team consisting of a general practitioner (GP), a lifestyle advisor (LSA), a physiotherapist and a dietician. The LSA (who may be a practice nurse or a physiotherapist) has the key role in this multidisciplinary team and offers wide-ranging lifestyle counselling aimed at promoting physical activity, improving diet and reducing psychological barriers by means of Motivational Interviewing [21]. A physiotherapist provides coaching for physical activity to enable participants to transfer to local exercise facilities, and a dietician provides advice on dietary improvement. The use of the 'BeweegKuur' programmes in primary care has proved to be feasible, as health care providers as well as participants are very positive about the programmes after having implemented it [23,24].

Care providers using 'BeweegKuur' offer three programmes, differing in the amount of support. The 'independent exercise programme' is intended for overweight and obese individuals without comorbidities, while the 'start-up exercise programme' and the 'supervised exercise programme' are both intended for adults with overweight or obesity who suffer from comorbidities or are morbidly obese. An earlier study showed that the amount of support required to achieve lifestyle changes increases with the severity of overweight and the presence of comorbidities [9]. Additionally, the number of feedback sessions is believed to be positively related to programme adherence [20]. Hence, the 'supervised exercise programme' involves more coaching by the physiotherapist.

Less intensively supervised programmes have been shown to be effective and cost-effective for people with type 2 diabetes or an increased risk of developing type 2 diabetes [8,13,17]. The effectiveness and cost-effectiveness of intensively supervised programmes for a population with very high weight-related health risk remain to be studied. Therefore, our randomized controlled trial aims to evaluate the effects of the 'supervised exercise programme', in terms of the amount of physical activity and related health risks, and its cost-effectiveness, compared to those of the 'start-up exercise programme', for this population. The time horizon of the study will be two years. The economic evaluation will involve cost-effectiveness and cost-utility analyses from a societal perspective. In addition, a process evaluation is planned.

## Methods/Design

### Study design

**Figure 1:**
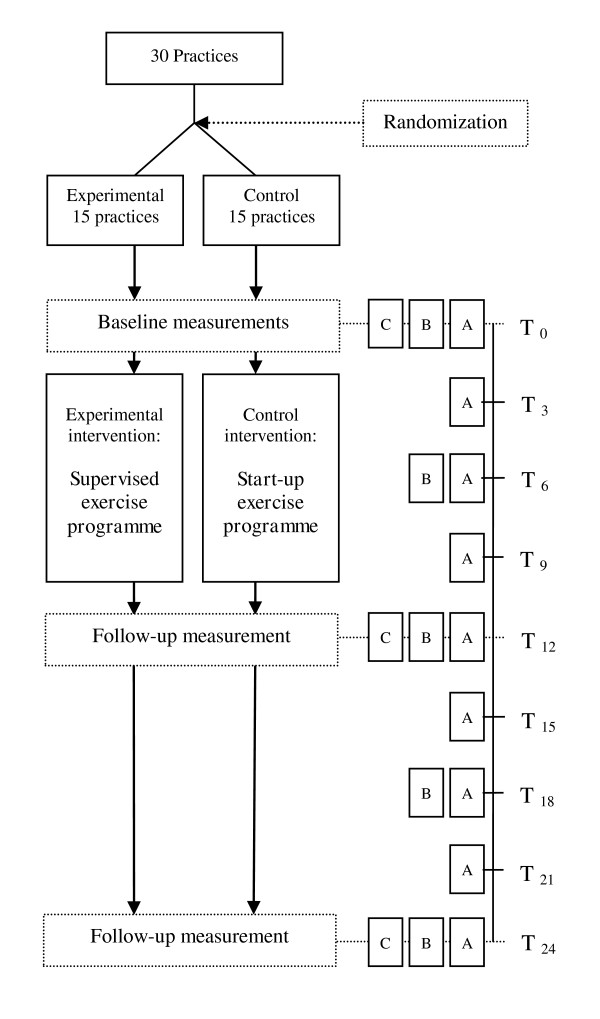
**Flowchart of the 'BeweegKuur' randomized controlled trial and measurements**. A. Health care utilisation and productivity losses, EQ5-D and process items in questionnaire every three months; B. Clinical outcomes measured in questionnaire every six months; C. Clinical outcome measurements performed every year. Table 2 shows outcomes in each category. T0-T24 represent moments of measurement. T0 = Baseline; T3 = Three months after baseline; T6 = Six months after baseline; ...; T24 = 24 months after baseline.

The present study is a clustered, multi-centre, randomized controlled trial evaluating the effectiveness and cost-effectiveness of the 'supervised exercise programme' versus the less intensively supervised 'start-up exercise programme' for patients with very high weight-related health risk. Thirty Dutch GP practices, each collaborating with a practice nurse, a physiotherapist and a dietician, have been randomly assigned to the control or experimental condition. In experimental practices, participants will take part in the 'supervised exercise programme', while participants in a control practice will take part in the 'start-up exercise programme'. Clinical outcome measurements take place at baseline, after 12 months (the end of the intervention period) and after 24 months (Figure 1). In addition, self-administered questionnaires comprising cost-, effect- and process-related outcome measures will be sent to the participants every three months.

This study is approved by the Medical Ethics Committee of the Maastricht University Medical Centre and is registered with Current Controlled Trials (ISRCTN46574304). The study is funded by The Netherlands Organization for Health Research and Development (ZonMW; project number: 123000002).

### Randomization

To reduce the risk of contamination between participants, and the risk of bias at the level of the professionals involved, entire practices have been allocated to the control or experimental condition. Prior to randomization, all practices have been matched pair wise based on size and location in an urban or rural area, to create two equivalent samples of 15 practices. In each pair, one practice has been randomized to the control condition, while the other was randomized to the experimental condition. To reduce the risk of contamination within a region, practices in the same region were allocated to the same condition as the first practice in that region that was randomized.

### Participants

Inclusion of participants started in July 2010. Inclusion criteria are (1) being overweight or obese (BMI 25-35 kg/m^2^) combined with the following serious related comorbidities: sleep apnoea, arthritis, cardiovascular disease and/or type 2 diabetes; or (2) being morbidly obese (BMI 35-40 kg/m^2^) but without these related serious comorbidities. In addition, participants should currently fail to meet the Dutch norm for healthy physical activity (30 minutes of moderate to vigorous physical activity on at least 5 days a week), have to be sufficiently motivated to change their physical activity level and dietary behaviour (to be judged subjectively by the LSA during intake) and have to give their informed consent.

Participants are being included via GPs, practice nurses and physiotherapists. The GP, practice nurse or physiotherapist selects patients by discussing the intervention during a consultation. However, they can also recruit patients actively (e.g. by searching the health care provider's records). The LSA screens the patients for eligibility. Exclusion criteria are serious mobility limitations precluding participation in the intervention programme, such as severe cardiac failure, serious angina pectoris and rheumatoid arthritis. Pregnancy is also an exclusion criterion. The GP decides whether patients should be excluded.

### Blinding

Although it is not possible to blind the professionals, randomization at the level of GP practices decreases the risk of contamination among the professionals. Participants are not aware of the allocation of their practice to the experimental or control condition.

To identify effects of observer bias, clinical measurements that might be affected (Åstrand test, Valk neuropathy test, body composition, hand grip strength and Timed Up and Go Test) will be repeated by a researcher blinded for the randomization of the practices and the baseline characteristics of the participants. Repeated measurements will be done for 20 participants in five randomly chosen control and five randomly chosen experimental practices.

### Intervention

After inclusion, participants have several consultations with the LSA, dietician and physiotherapist during a one-year intervention period. The number of consultations differs per programme (table 1).

**Table 1:** Contents and number of consultations planned in the control and experimental interventions

Experimental intervention:		Control intervention:	
the 'supervised exercise programme'		the 'start-up exercise programme'	
***Contents of LSA consultations***	***No. of meetings***	***Contents of LSA consultations***	***No. of meetings***
Intake	1	Intake	1
Guidance and follow-up	5	Guidance and follow-up	5

***Contents of dietician consultations ***	***No. of meetings***	***Contents of dietician consultations ***	***No. of meetings***
Intake	1	Intake	1
Guidance and follow-up		Guidance and follow-up	
- Individual	2	- Individual	2
- Group	7	- Group	7

***Contents of physiotherapist consultations***	***No. of meetings***	***Contents of physiotherapist consultations***	***No. of meetings***
Intake	1	Intake	1
Setting up exercise plan	2	Setting up exercise plan	1
Supervised exercise	26 - 34	Follow-up	4
Follow-up	3 - 4		

#### *Experimental intervention ('supervised exercise programme')*

The LSA has a key role in supporting the participants and is the point of contact between the participants and the other health care providers in the 'BeweegKuur' programme. Following an individual intake to set personal goals, participants will have five individual consultations with the LSA to discuss progress in terms of behavioural change, roughly once every 10 weeks, during the one-year intervention period. Consultations with the dietician will consist of nutritional recommendations, education, coping with high-risk situations, checking dietary behaviour and fellow-sufferer contact. Advice will be based on various Dutch guidelines for diabetes, overweight and obesity [25-27]. After an individual intake session by the dietician, seven group sessions are planned. The group meetings comprise guidance and advice by the dietician and are scheduled throughout the year. In addition, two individual follow-up meetings are planned during the intervention period to prevent relapse.

The physiotherapist will provide coaching to enable participants to exercise unsupervised in local exercise facilities. Coaching by the physiotherapist will be initiated by setting out personal goals and identifying barriers hampering engagement in physical activity. Coaching will consist of supervised exercise to overcome any barriers identified and increase physical capacity. Two or three sessions of supervised exercise per week will be planned over a period of 12 weeks. After these 12 weeks, the physiotherapist will evaluate whether the participant is able to exercise without supervision. The coaching period can be extended by four weeks if the participant does not seem able to exercise independently in local facilities. In all, the physiotherapist's coaching will take 12 to 16 weeks. After coaching by the physiotherapist has ended, the five follow-up consultations with the LSA and three or four follow-up consultations with the physiotherapist are planned, to help participants adopt and continue independent exercise activities. Both the LSA and the physiotherapist will help the participant find suitable existing exercise facilities during the entire intervention period.

#### *Control intervention ('start-up exercise programme')*

The number of consultations and the characteristics of the guidance provided by the LSA and the dietician in the control condition are the same as in the experimental condition. However, participants of the 'start-up exercise programme' will only have six consultations with the physiotherapist, which are planned during the first two months of the intervention period. The consultations with the physiotherapist consist of identifying barriers to physical activity and drawing up a plan to remain physically active without supervision by health care providers. If deemed necessary by the physiotherapist, participants can exercise under supervision during these meetings to overcome barriers to physical activity. Progress and complications relating to the exercise plan will be discussed in consultations with the physiotherapist from approximately two months after the start of the intervention. Additionally, participants will be coached in the adoption and maintenance of independent exercise activities during the five follow-up consultations with the LSA during and after the two months of guidance by the physiotherapist.

### Outcomes

Clinical assessments will be done by the 'BeweegKuur' health care providers (LSA or physiotherapist) in their own practice. After the baseline measurement, three different measurement intervals will be used, depending on the variable to be measured: (A) 3 months, (B) 6 months and (C) 12 months (figure 1 and table 2).

**Table 2:** Type of outcomes in each measurement category (time intervals are shown in figure 1)

A. Costs, utility and process assessment (self-administered questionnaires)	B. Clinical outcomes (self-administered questionnaires)	C. Clinical outcomes (measurements by professionals)
- Health care use, other expenses and productivity losses	- Physical activity	- Physical activity by accelerometry
- Quality of life	- Dietary behaviour	- Risk factors for comorbidities
- Process evaluation		- Physical fitness

### Physical activity

The primary outcome of this study will be the amount of physical activity that participants engage in, as measured by means of accelerometry and the short version of the International Physical Activity Questionnaire (IPAQ). The IPAQ short form will be included in the participants' questionnaire every six months, and consists of questions concerning the time spent on physical activity at specific intensities and the number of days on which this happened. Median values of activity categories will be calculated and expressed as metabolic equivalent (MET) minutes per week. The self-administered IPAQ short form has been reported to be sufficiently valid and reliable for use in developed countries [28].

Accelerometry offers an objective way to assess physical activity. The CAM is a tri-axial accelerometer developed and manufactured by Maastricht Instruments. The CAM software is able to distinguish between sedentary behaviour, standing and activity, and has been validated for adults in laboratory conditions [29]. The device weighs approximately 100 g (63 × 45 × 18 mm) and its sampling frequency is 25 Hz. Participants will wear the CAM for four consecutive days; data from waking up until going to sleep will be used for analysis. Because the CAM is not waterproof, participants will have to remove the CAM for swimming, showering, bathing etc. and will be asked to write down their activities in a diary for such non-wearing intervals. The main outcomes of the CAM measurements will be the amount of moderate to vigorous physical activity (MVPA) and the amount of time spent sedentary, standing or active.

### Dietary habits

The short version of the Fat Food questionnaire will be included in the questionnaire sent to the participants every six months [30]. The length of this validated Fat Food questionnaire has been reduced to maximize the number of questions completed. Twenty-one items address the respondent's regular eating pattern (e.g. consumption of vegetables, lettuce and fruit) and consumption of high-fat meals (e.g. take-away food), snacks and candy.

### Risk factors for comorbidities

Body composition (weight, fat mass and fat-free mass) will be measured with a tetrapolar bioelectrical impedance device (OMRON BF511). Blood samples will be taken to analyse fasting glucose (mmol/L), HbA1c (% or mmol/L), total cholesterol (mmol/L), HDL (mmol/L), triglycerides (mmol/L) and creatinin (μmol/L). Systolic and diastolic blood pressure, resting heart rate and BMI will also be measured.

### Physical fitness

Peripheral neuropathy is related to functional capacity in type 2 diabetes patients [31]. Diabetic neuropathy will be examined using the Valk neuropathy test [32], while hand grip strength [33,34] and the timed 'up and go' test will be used as measures of functional capacity [35]. To familiarize participants with the Borg scale, it will be used during the timed 'up and go' and hand grip strength tests.

The participant's aerobic capacity will be estimated using the sub-maximal Åstrand cycle test [36,37]. The Åstrand cycle test will always be administered by the physiotherapists at their own practice. Participants will start cycling at 50 Watt at a pedal rate of 50 revolutions per minute. The test will be conducted at heart rates between 120 and maximum heart rate. The heart rates of the fifth and sixth minutes of the test will be recorded and used to estimate the aerobic capacity from a nomogram [36]. This estimated aerobic capacity will be corrected for age [38]. In addition, the rate of perceived exertion will be recorded at each work level by means of the Borg score, with a range of 6-20. Participants whose heart rate cannot be used as a reference for physical fitness (e.g. patients who use beta blockers) will do the Åstrand cycle test with a Borg score between 13 and 17 [39].

### Economic evaluation

The economic evaluation will compare costs and effects of the 'supervised exercise programme' with those of the 'start-up exercise programme'. The economic evaluation will be performed from a societal perspective, which implies that all relevant costs and outcomes will be taken into account, regardless of who pays the costs and who benefits from the effects. A time horizon of two years will be used.

Both a cost-effectiveness analysis (CEA) and a cost-utility analysis (CUA) will be done. The CEA will present clinical outcomes in terms of physical activity measured by means of accelerometry and the short version of the IPAQ [28]. The CUA will present effects in terms of quality-adjusted life years (QALYs) measured by means of the internationally developed EuroQol [40] in three-monthly self-administered questionnaires. The QALY incorporates multiple aspects of the intervention (e.g. side-effects) and allows comparisons among different (lifestyle) interventions in different target populations. A direct value for each state of health will be generated using social tariff, which involves an algorithm for interpolating EuroQol outcomes into population utilities based on the United Kingdom valuation [41] and the Dutch valuation [42].

Programme costs, health care costs, patient and family costs as well as loss of productivity will be assessed. Volumes of healthcare use, loss of productivity and other expenses will be identified by means of three-monthly self-administered questionnaires. Cost valuation will use the Dutch manual for cost analysis in health care research [43], while real costs will be used otherwise. Cost prices will be expressed in Euros from the baseline year 2011, and otherwise indexed to the baseline year, as suggested in the Dutch manual [44]. Because the recruitment period will be 12 months and the follow-up period 24 months, costs and effects in the second year of follow-up will be discounted.

### Process evaluation

A process evaluation will be used to gain insight into reach and the attendance rates of the target population, implementation fidelity, delivered intervention dose, and participant perception of the intervention [45] in order to support the interpretation of the effects. The process evaluation will assess personal factors of participants (e.g. self-efficacy, motivation towards being physically active and eating healthy), self-report environmental variables as used in the International Physical Activity Prevalence Study (e.g. presence of pavements and perceived neighbourhood safety) [46], number and duration of the consultations with the health care providers involved, satisfaction with the intervention contents and feasibility of the intervention. The process will be evaluated by means of self-administered questionnaires for participants, with closed and open-ended questions. In addition, registration forms, short surveys and semi-structured interviews with the relevant professionals in each practice will assess relevant barriers and facilitators for intervention implementation. Adverse events will be recorded.

### Sample size

The intended sample size is based on the amount of MVPA in minutes per week. An increase of 50 minutes of MVPA per week by participants in the 'supervised exercise programme', as compared to participants in the 'start-up exercise programme' will be considered a clinically meaningful increase in MVPA. The standard deviation of MVPA in this population has been reported to be 120 minutes/week [47]. A sample size of 91 participants per condition will be needed to detect a difference of 50 minutes of MVPA per week, with 80% power and 5% significance (two-sided). Assuming a drop-out rate of 30%, this would require 119 participants in each programme, i.e. 238 participants in total.

Allocation to the conditions, however, will take place at the level of GP practices, so clustering of patients within these practices should be taken into account. Assuming an intra-cluster correlation of 5%, and a total of 20 participants per practice, a total sample of 24 practices (n = 480) will be needed. As practices may also drop out of this study, we will include and allocate an additional six practices to account for this potential drop-out. The choice of six practices is completely arbitrary. This results in a projected total sample of 600 participants divided over 30 practices.

### Analysis

Baseline characteristics (BMI, age, gender, amount of MVPA by accelerometry) of both participants and clusters will be analysed by means of descriptive statistics. Statistical analyses will be performed according to the intention-to-treat principle, while additional analyses will be done using the per-protocol principle.

Differences between outcomes in the control and intervention groups at different time points will be assessed using multi-level analyses. This type of analysis takes into account the longitudinal nature of the data, as well as the impact of cluster randomization.

Differences in costs and effects will be presented as incremental cost-effectiveness ratios (ICERs). ICERs represent the differences in mean costs between the experimental and control interventions in the numerator and the difference in mean effects between the two groups in the denominator. Sensitivity analysis will be used to assess the robustness of the assumptions made in our base case analysis.

Outcomes of the process evaluation will be studied by means of descriptive statistics.

## Discussion

The aim of this study is to determine whether the 'supervised exercise programme' of the 'BeweegKuur' intervention has positive effects on physical activity levels compared to the less intensively supervised 'start-up exercise programme' in a population of overweight and obese adults with very high weight-related health risk, and to assess the difference in costs involved between the two lifestyle programmes. The risk of chronic diseases is known to decrease if overweight or obese persons achieve a more physically active lifestyle. This might also reduce health care expenses. Therefore, an improved lifestyle resulting from an intervention like 'BeweegKuur' is expected to have major positive consequences at both individual and societal level.

Increasing adherence to lifestyle interventions is crucial. The proposed solutions (Motivational Interviewing and patient-specific guidance) might decrease the drop-out rate, thereby increasing the possible effects of the intervention and decreasing health care expenses. Nevertheless, these solutions require extra time investment by health care providers, raising intervention costs, so examining the cost-effectiveness of lifestyle interventions in primary care is of great social interest. The economic evaluation will provide insight into the cost-effectiveness regarding the effects on quality of life and physical activity, to support decisions concerning insurance coverage of the 'BeweegKuur' intervention and similar lifestyle interventions.

Objectively measuring physical activity levels enables accurate conclusions to be drawn about the direct effects of the intervention. Moreover, this will afford new insights into physical activity and inactivity patterns in an overweight population with very high weight-related health risk.

This study aims to gain insight into the cost-effectiveness of the 'supervised exercise programme' compared to the 'start-up exercise programme', in order to inform decision and policy makers about the implementation of 'BeweegKuur' in primary care in the Netherlands. In addition, the process evaluation will provide detailed information about the feasibility of implementing these two interventions and the degree of satisfaction of participants, and will also provide some insight into the mechanisms by which the components of the intervention exert their effects.

## Competing interests

The authors declare that they have no competing interests.

## Authors' contributions

BB is the investigator for the effect evaluation and wrote most of the present manuscript. MH is the investigator for the economic evaluation, wrote the design of the economic evaluation and helped draft the present manuscript. EV contributed to the design of the trial and supervises the methodology. NS supervises the clinical part of the study. SK developed the design of the trial and provides advice on process evaluation. HS is the principal investigator, developed the design of the study. All authors have read and approved the final version of the manuscript.

## Pre-publication history

The pre-publication history for this paper can be accessed here:

http://www.biomedcentral.com/1471-2458/11/815/prepub
